# Prognostic Role of Uric Acid-to-Albumin Ratio in Patients with Metastatic Breast Cancer Treated with CDK4/6 Inhibitors

**DOI:** 10.3390/jcm15103850

**Published:** 2026-05-16

**Authors:** Talat Aykut, Mehmet Zahid Koçak, Oğuzhan Yıldız, Bahattin Engin Kaya, Ali Fuat Gürbüz, Ömer Genç, Melek Karakurt Eryılmaz, Murat Araz, Mehmet Artaç

**Affiliations:** Department of Medical Oncology, School of Medicine, Necmettin Erbakan University, Konya 42080, Turkey; mehmetzahidkocak@hotmail.com (M.Z.K.); dr.oguzhan@outlook.com (O.Y.); md.enginkaya@gmail.com (B.E.K.); dr.alifuatgurbuz@hotmail.com (A.F.G.); omergenc58@hotmail.com (Ö.G.); drangelkarakurt@hotmail.com (M.K.E.); zaratarum@yahoo.com (M.A.); mehmetartac@yahoo.com (M.A.)

**Keywords:** metastatic breast cancer, CDK4/6 inhibitor, uric acid to albumin ratio, prognosis, survival

## Abstract

**Background/Objectives**: The prognostic significance of the uric acid-to-albumin ratio (UAR) in patients with hormone receptor-positive (HR+) and human epidermal growth factor receptor 2-negative (HER2−) metastatic breast cancer treated with cyclin-dependent kinase 4/6 (CDK4/6) inhibitors has not been adequately investigated. This study aimed to investigate the association between baseline UAR and survival outcomes in this patient population. **Methods**: This retrospective study included HR-positive/HER2-negative metastatic breast cancer patients treated with ribociclib or palbociclib at Necmettin Erbakan University between May 2020 and April 2025. UAR was calculated by dividing the serum uric acid level (mg/dL) by the serum albumin level (g/dL). Based on receiver operating characteristic (ROC) analysis, the optimal cut-off value for UAR was identified as 1.0 (AUC = 0.67; sensitivity 68%; specificity 58%). Patients were subsequently classified into two groups as UAR < 1 and UAR ≥ 1. Progression-free survival (PFS) and overall survival (OS) were estimated using the Kaplan–Meier method and compared with the log-rank test. Independent prognostic factors were evaluated using Cox regression analyses. **Results**: A total of 118 eligible patients were included in the analysis, including 34 (28.8%) in the UAR < 1 group and 84 (71.2%) in the UAR ≥ 1 group. The proportion of postmenopausal patients was significantly higher in the UAR ≥ 1 group (*p* = 0.01). Kaplan–Meier analysis showed that median PFS was not reached in the UAR < 1 group, whereas it was 33.05 months in the UAR ≥ 1 group (log-rank *p* = 0.06). Median OS was not reached in the UAR < 1 group and was 50.7 months in the UAR ≥ 1 group (*p* = 0.017). Multivariate Cox regression analysis demonstrated that UAR < 1 was associated with improved PFS (HR = 0.65; 95% CI: 0.34–0.89; *p* = 0.04). Postmenopausal status emerged as an independent adverse prognostic factor for PFS (HR = 1.92; 95% CI: 1.10–4.05; *p* = 0.04). In addition, UAR < 1 was associated with a reduced risk of mortality in the OS analysis (HR = 0.61; 95% CI: 0.26–0.87; *p* = 0.01). **Conclusions**: Lower baseline UAR was associated with more favorable survival outcomes in HR-positive/HER2-negative metastatic breast cancer patients treated with CDK4/6 inhibitors. As an inexpensive and easily accessible biomarker derived from routine laboratory parameters, UAR may provide additional prognostic information for clinical risk stratification.

## 1. Introduction

Breast cancer is the most frequently diagnosed malignancy among women worldwide and remains one of the leading causes of cancer-related mortality [1]. Hormone receptor-positive (HR+) and human epidermal growth factor receptor 2-negative (HER2–) tumors represent the predominant molecular subtype, accounting for approximately 60–70% of all breast cancer cases [2]. Over the past decade, the integration of cyclin-dependent kinase 4/6 (CDK4/6) inhibitors with endocrine therapy has emerged as the standard approach for the treatment of this patient group in the metastatic stage. Pivotal phase III clinical trials involving palbociclib, ribociclib and abemaciclib have demonstrated substantial improvements in both progression-free and overall survival [3,4,5]. However, clinical responses to treatment are highly heterogeneous, and comparable clinical benefit is not observed in all patients owing to intrinsic or acquired resistance mechanisms. Despite these advances, resistance to CDK4/6 inhibitors eventually develops in a substantial proportion of patients. Consequently, there is an increasing need for biomarkers that are feasible for routine clinical implementation and capable of predicting prognosis and guiding individualized treatment strategies.

Emerging evidence increasingly indicates that cancer progression is influenced not only by intrinsic tumor biology but also by systemic inflammation, metabolic status, and nutritional reserve [6]. In this context, the role of uric acid in cancer biology exhibits a remarkable dual structure. Uric acid, which has antioxidant properties at physiological levels, may act as a pro-inflammatory signaling molecule in the tumor microenvironment by triggering cellular stress responses at high concentrations [7]. Experimental studies suggest that increased uric acid levels may affect cell cycle regulation and enhance proliferative activity in breast cancer cells via AhR/p27Kip1/cyclin E1 signaling pathways [8]. In contrast, serum albumin is considered a negative acute phase reactant, reflecting nutritional status as well as systemic inflammatory burden. Reduced albumin levels are associated with impaired nutritional status and heightened inflammatory activity, which may negatively impact the tolerability and effectiveness of systemic anticancer therapies [9].

The complementary biological features of uric acid and albumin provide a strong rationale for their combined clinical evaluation. The uric acid-to-albumin ratio (UAR) has been defined as a pragmatic biomarker integrating metabolic stress, systemic inflammation, and nutritional status into a single composite measure. Previous studies have demonstrated the prognostic utility of UAR across a range of clinical conditions, including cardiovascular diseases, sepsis and multiple solid malignancies [10,11,12]. However, data regarding the prognostic relevance of UAR in metastatic breast cancer patients treated with CDK4/6 inhibitors remain limited. Accordingly, the present study aimed to evaluate the prognostic impact of baseline pre-treatment UAR on progression-free and overall survival in patients with HR+ HER2- metastatic breast cancer receiving CDK4/6 inhibitor therapy.

## 2. Materials and Methods

**Figure 1:**
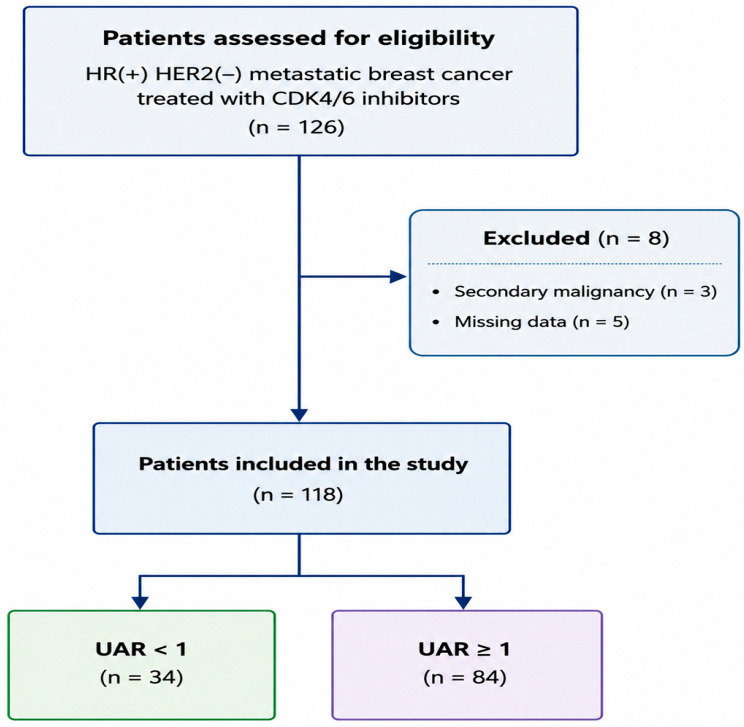
Flow chart of the study.

A total of 126 patients with HR-positive, HER2-negative metastatic breast cancer treated with CDK4/6 inhibitors (ribociclib or palbociclib) at Necmettin Erbakan University between May 2020 and April 2025 were initially screened for eligibility. Patients aged ≥18 years with histopathologically confirmed HR-positive/HER2-negative metastatic breast cancer who received ribociclib or palbociclib were eligible for inclusion. Patients with secondary malignancies (*n* = 3) or incomplete data (*n* = 5) were excluded (Figure 1).

The study was conducted in accordance with the Declaration of Helsinki and approved by the Necmettin Erbakan University Ethics Committee (application no: 29105, decision no: 2026/6288). Medical records were retrospectively reviewed to obtain demographic and clinical data, including clinical stage, comorbidities, menopausal status, and metastatic sites. Baseline laboratory parameters measured before treatment initiation included albumin, uric acid, creatinine, hemoglobin, leukocyte count, and other routine biochemical markers. The primary predictive parameter of the study, the uric acid-to-albumin ratio (UAR), was calculated by dividing the serum uric acid level (mg/dL) by the serum albumin level (g/dL). Based on receiver operating characteristic (ROC) curve analysis and previous literature, the optimal cut-off value for UAR was determined as 1.0 (AUC = 0.67, sensitivity = 68%, specificity = 58%). Patients were subsequently stratified into two groups according to UAR values (UAR < 1 and UAR ≥ 1). Variables assessed included age (<65 years vs. ≥65 years), menopausal status (premenopausal vs. postmenopausal), presence of comorbidities, metastatic disease at diagnosis, bone metastasis, laboratory parameters (albumin, C-reactive protein, uric acid, creatinine, urea, hemoglobin, white blood cell count, platelet count), type of CDK4/6 inhibitor administered (ribociclib, palbociclib), best treatment response (complete response, partial response, stable disease, or progression), and presence of Grade 3–4 toxicities. Treatment response was assessed based on radiological and clinical evaluation during routine follow-up. Adverse events were graded according to the Common Terminology Criteria for Adverse Events (CTCAE). Progression-free survival (PFS) was calculated from the initiation of CDK4/6 inhibitor therapy to radiological or clinical disease progression, or death from any cause. Overall survival (OS) was calculated from treatment initiation to death from any cause.

### Statistical Analysis

Statistical analyses were conducted using IBM SPSS Statistics version 26.0 (IBM Corp., Armonk, NY, USA). Categorical variables are presented as frequencies (*n*) and percentages (%), whereas continuous variables are expressed as mean ± standard deviation. Comparisons between UAR groups were performed using the chi-square test or Fisher’s exact test for categorical variables. Continuous variables with normal distribution were analyzed using the Independent Samples t-test, while the Mann–Whitney U test was used for non-normally distributed variables. PFS and OS were estimated using the Kaplan–Meier method and compared with the log-rank test. Univariate Cox regression analyses were initially performed to assess factors potentially associated with PFS and OS. Variables showing clinical relevance or a *p*-value <0.10 in univariate analysis were subsequently included in the multivariate Cox regression model. Variable selection was performed using the Likelihood Ratio method. Results are presented as hazard ratios (HRs) with 95% confidence intervals (CIs). A two-sided *p*-value <0.05 was considered statistically significant.

## 3. Results

A total of 118 patients with hormone receptor-positive (HR+) and human epidermal growth factor receptor 2-negative (HER2-negative) metastatic breast cancer were included in the analysis. Baseline demographic, clinical, and laboratory characteristics according to UAR groups are summarized in Table 1. Of these patients, 34 (28.8%) were classified in the low UAR group (UAR < 1) and 84 (71.2%) in the high UAR group (UAR ≥ 1).

Comparative analysis showed a statistically significant difference in menopausal status between the groups (*p* = 0.01), with a markedly higher proportion of postmenopausal patients observed in the high UAR group (71.4%). Age (*p* = 0.087) and presence of comorbidities (*p* = 0.06) demonstrated borderline statistical significance, with higher frequencies of patients aged ≥ 65 years (26.2%) and those with comorbid conditions (39.3%) in the UAR ≥1 group.

Regarding laboratory parameters, serum albumin levels tended to be higher in the UAR <1 group (4.44 ± 0.32 g/dL) compared with the UAR ≥1 group (4.27 ± 0.40 g/dL), approaching statistical significance (*p* = 0.07). Body weight also showed a borderline increase in the UAR ≥1 group (75.3 ± 16.9 kg; *p* = 0.06). No significant differences were identified between the two groups with respect to other laboratory parameters, including C-reactive protein, uric acid, creatinine, hemoglobin, and white blood cell count (WBC), nor in clinical characteristics such as metastatic burden, type of CDK4/6 inhibitor administered, treatment response, or toxicity.

**Figure 2:**
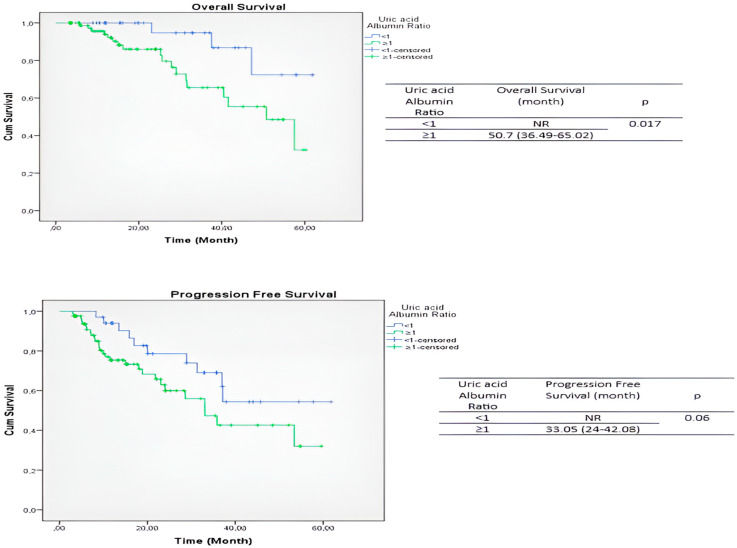
Kaplan–Meier curves for progression-free survival (PFS) and overall survival (OS) according to uric acid-to-albumin ratio (UAR).

Kaplan–Meier survival analysis of the entire cohort demonstrated that the median progression-free survival (PFS) was not reached in patients with UAR < 1, whereas patients with high UAR exhibited a median PFS of 33.05 months (95% CI: 24–42.08), approaching statistical significance (*p* = 0.06) (Figure 2). In contrast, a statistically significant difference in overall survival (OS) was observed between the two groups. Median OS was not reached in the low UAR group, while patients with UAR ≥ 1 had a median OS of 50.7 months (95% CI: 36.49–65.02) (*p* = 0.017) (Figure 2).

In the univariate Cox regression analysis (Table 2), a borderline significant association was observed between low UAR (<1) and improved PFS (HR = 0.50, 95% CI: 0.24–1.05, *p* = 0.06). Among the other variables, only menopausal status showed a trend toward statistical significance, although this did not reach significance (HR = 1.46 for premenopausal status, *p* = 0.23). In the multivariate analysis, variables considered clinically important or approaching significance in univariate analysis, including age, comorbidity, menopausal status and UAR, were subsequently incorporated into the model. After adjustment, low UAR (<1) emerged as an independent predictor of prolonged PFS (HR = 0.65, 95% CI: 0.34–0.89, *p* = 0.04). Additionally, postmenopausal status was independently associated with poor PFS outcomes (HR = 1.92, 95% CI: 1.10–4.05, *p* = 0.04).

For overall survival, the univariate analysis (Table 3) demonstrated a statistically significant protective effect of low UAR (<1) on OS (HR = 0.72, 95% CI: 0.17–0.96, *p* = 0.02). Elevated serum uric acid levels also showed a borderline association with worse OS (HR = 1.55, *p* = 0.08). In the multivariate analysis, variables that were significant or near significant in the univariate analysis, including UAR, uric acid, comorbidities, and bone metastasis, were incorporated into the model. The final model confirmed low UAR (<1) as a strong and independent protective prognostic factor for OS (HR = 0.61, 95% CI: 0.26–0.87, *p* = 0.01). No other variables demonstrated independent statistical significance.

## 4. Discussion

In this retrospective study, the uric acid-to-albumin ratio (UAR) calculated prior to CDK4/6 inhibitor therapy was shown to be associated with survival outcomes in patients with HR-positive and HER2-negative metastatic breast cancer. Lower baseline UAR levels were associated with a more favorable prognosis in terms of both progression-free survival (PFS) and overall survival (OS). Notably, the persistence of UAR as an independent prognostic factor for OS in multivariate analyses highlights the potential clinical relevance of this parameter.

Although the association between UAR and PFS demonstrated borderline significance in the Kaplan–Meier analyses, it is noteworthy that it became statistically significant in the multivariate analyses. In metastatic breast cancer, OS represents a more comprehensive endpoint, reflecting not only the early disease progression related to treatment but also the post-progression period and the cumulative impact of successive lines of therapy. Therefore, the stronger association observed between UAR and OS is consistent with the influence of patients’ systemic condition on long-term outcomes.

UAR is a composite biomarker that reflects systemic inflammation, metabolic stress, and nutritional status simultaneously. Although uric acid may exhibit antioxidant properties under physiological conditions, elevated levels have also been associated with inflammatory signaling and proliferative activity within the tumor microenvironment. Albumin, on the other hand, serves as an indirect indicator of nutritional status and chronic inflammation, with low levels associated with adverse clinical outcomes. The combined assessment of these two parameters may, therefore, provide a more comprehensive reflection of a patient’s biological condition than either measurement alone [13,14].

Various indices reflecting systemic inflammation have been described in the literature, with commonly used examples including the neutrophil-to-lymphocyte ratio, platelet-to-lymphocyte ratio, and C-reactive protein-to-albumin ratio [15]. These indices predominantly capture a single dimension of the inflammatory response. In contrast, the uric acid-to-albumin ratio incorporates both uric acid, representing metabolic burden, and albumin, reflecting nutritional status and inflammation, thereby offering the potential for a more holistic evaluation of the patient’s biological status [16]. Although these inflammatory scores were not analyzed in the present study, UAR may conceptually be considered as a complementary biomarker to these indices.

The clinical relevance of UAR is not limited to oncological diseases. UAR has also been reported to be associated with prognosis in non-oncological clinical conditions such as cardiovascular diseases and sepsis [10,11,12,13]. These findings suggest that UAR may serve as a general host-related biomarker reflecting systemic inflammation and metabolic burden. However, within the oncological context, the clinical significance of UAR should be interpreted in relation to tumor biology and metastatic behavior [17].

Recent studies have increasingly highlighted the role of UAR in solid tumors. In a retrospective analysis involving patients with colorectal cancer, elevated UAR levels were found to be significantly associated with the presence of bone metastases and demonstrated acceptable diagnostic accuracy in predicting metastatic disease [18]. Similarly, a retrospective study conducted in patients with surgically treated non-small cell lung cancer reported that higher preoperative UAR levels were associated with occult lymph node metastasis [12]. These findings support the notion that UAR may be linked to tumor aggressiveness and metastatic potential across various solid malignancies, thereby complementing the survival associations observed in metastatic breast cancer in the present study.

An important finding of the present study was the identification of postmenopausal status as an independent negative prognostic factor for progression-free survival. It may be hypothesized that hormonal and metabolic changes occurring in the postmenopausal period influence tumor biology through alterations in uric acid metabolism and albumin levels [19]. The tendency toward higher body weight observed in the high UAR group further suggests that obesity-related chronic inflammation may contribute to prognosis via metabolic parameters [20].

A key clinical advantage of UAR is its ease of calculation using routine biochemical tests without additional cost. This characteristic makes UAR a practical parameter that may contribute to risk stratification in patients with metastatic breast cancer scheduled to receive CDK4/6 inhibitor therapy. However, UAR should not be considered a determinant guiding treatment selection, but rather a complementary indicator supporting follow-up strategies and supportive care planning.

This study has several limitations. The retrospective and single-center design may introduce selection bias. The relatively limited sample size may have reduced the statistical power of certain subgroup analyses. Furthermore, UAR was evaluated at a single pre-treatment time point, precluding evaluation of dynamic changes during therapy. In addition, detailed data regarding prior adjuvant endocrine therapy, hormone replacement therapy, chemotherapy, or radiotherapy exposure were not consistently available in the retrospective records. Therefore, the potential impact of previous treatments on UAR levels could not be fully evaluated. Nevertheless, the homogeneous patient population and the use of multivariate analyses to control for potential confounding factors strengthen the reliability of the findings. Future prospective multicenter studies incorporating serial UAR measurements may further clarify the prognostic and clinical utility of UAR in metastatic breast cancer.

## 5. Conclusions

This study demonstrated that pre-treatment UAR was significantly associated with survival outcomes in patients with HR-positive and HER2-negative metastatic breast cancer treated with CDK4/6 inhibitors. Lower UAR levels were associated with a more favorable clinical course, suggesting that UAR may serve as a practical biomarker reflecting systemic inflammation, metabolic status, and nutritional reserve, thereby contributing to the risk stratification.

The ease of calculating UAR using routine biochemical tests without additional cost represents a notable advantage for clinical practice. Nevertheless, larger prospective multicenter studies are needed to confirm these findings and better define the potential clinical role of UAR.

## Figures and Tables

**Table 1:** Baseline patient characteristics according to uric acid-to-albumin ratio (UAR).

Features (*n*)		Uric Acid-to-Albumin Ratio		*p*
		<1	≥1	
Age	<65	30 (88.2%)	62 (73.8%)	0.08
	≥65	4 (11.8%)	22 (26.2%)	
Comorbidity	Absent	29 (85.3%)	51 (60.7%)	0.06
	Present	5 (14.7%)	33 (39.3%)	
Body Mass Index (kg/m^2^)	<25	11 (32.4%)	19 (22.6%)	0.08
	25–29.9	18 (52.9%)	35 (41.7%)	
	≥30	5 (14.7%)	30 (35.7%)	
Menopausal status	Premenopause	23 (67.6%)	24 (28.6%)	0.01
	Postmenopause	11 (32.4%)	60 (71.4%)	
Metastasis at diagnosis	Absent	18 (52.9%)	37 (44.0%)	0.38
	Present	16 (47.1%)	47 (56.0%)	
Grade 3–4 toxicity	Absent	24 (70.6%)	61 (72.6%)	0.80
	Present	10 (29.4%)	21 (25.0%)	
Local recurrence	Absent	33 (97.1%)	75 (89.3%)	0.17
	Present	1 (2.9%)	9 (10.7%)	
CDK4/6 inhibitor	Ribociclib	28 (82.4%)	68 (81.0%)	0.86
	Palbociclib	6 (17.6%)	16 (19.0%)	
Best CDK4/6 response	Complete response	4 (11.8%)	3 (3.6%)	0.11
	Partial response	22 (64.7%)	63 (75.0%)	
	Stable disease	8 (23.5%)	13 (15.5%)	
	Progression	0 (0%)	5 (6.0%)	
Albumin (g/dL)		4.44 ± 0.32	4.27 ± 0.40	0.07
CRP (mg/L)		15.07 ± 9.67	13.10 ± 30.19	0.52
Uric acid (mg/dL)		5.31 ± 0.59	5.70 ± 1.03	0.82
Creatinine (mg/dL)		0.67 ± 0.13	0.65 ± 0.18	0.20
Urea (mg/dL)		35.7 ± 8.2	31.0 ± 9.9	0.70
Height (cm)		158.2 ± 7.1	159.9 ± 6.2	0.22
Weight (kg)		66.4 ± 9.0	75.3 ± 16.9	0.06
WBC (×10^9^/L)		6.29 ± 1.80	7.01 ± 2.10	0.08
Lymphocyte (×10^9^/L)		1.78 ± 0.61	1.86 ± 0.61	0.51
Hemoglobin (g/dL)		12.4 ± 1.54	12.98 ± 1.60	0.07
Platelet (×10^3^/µL)		313.823 ± 90.045	291.53 ± 99.24	0.25

**Table 2:** Univariate and multivariate analyses of predictive factors for progression-free survival (PFS).

Progression Free Survival Features		Study Population					
		Univariate			Multivariate		
		Hazard Ratio	95% Confidence Interval	*p*	Hazard Ratio	95% Confidence Interval	*p*
Age	≤65 vs. ≥65	1.59	0.66–3.81	0.29	1.99	0.73–5.39	0.17
Comorbidity	Yes vs. No	0.92	0.47–1.79	0.81	1.00	0.48–2.06	0.98
Body Mass Index (kg/m^2^)	<25	Reference		0.57	-	-	-
	25–29.9	0.88	0.30–1.15		-	-	-
	≥30	0.98	0.40–1.26		-	-	-
Menopausal status	Pre vs. Post	1.46	0.77–2.77	0.23	1.92	1.10–4.05	0.04
Metastasis at diagnosis	Yes vs. No	1.10	0.58–2.09	0.76	-	-	-
Bone metastasis	Yes vs. No	0.83	0.36–1.89	0.66	-	-	-
Uric acid-to-albumin ratio	<1 vs. ≥1	0.50	0.24–1.05	0.06	0.65	0.34–0.89	0.04
Hemoglobin		0.94	0.77–1.15	0.59	-	-	-
Albumin		0.95	0.88–1.04	0.32	-	-	-
Creatinine		0.75	0.65–1.68	0.41	-	-	-
Uric acid		1.18	0.94–1.50	0.14	1.12	0.76–1.64	0.56

**Table 3:** Univariate and multivariate analyses of predictive factors for overall survival (OS).

Overall Survival		Study Population					
		Univariate			Multivariate		
		Hazard Ratio	95% Confidence Interval	*p*	Hazard Ratio	95% Confidence Interval	*p*
Age	<65 vs. ≥65	0.79	0.29–2.16	0.65	-	-	-
Comorbidity	Yes vs. No	1.02	0.41–2.53	0.96	1.47	0.58–3.76	0.42
Body Mass Index (kg/m^2^)	<25	Reference		0.71	-	-	-
	25–29.9	0.97	0.32–2.93	0.96	-	-	-
	≥30	0.67	0.21–2.13	0.50	-	-	-
Menopausal status	Pre vs. Post	1.09	0.46–2.59	0.83	1.25	0.51–3.04	0.63
Metastasis at Diagnosis	Yes vs. No	0.81	0.34–1.93	0.63	-	-	-
Bone Metastasis	Yes vs. No	1.64	0.66–4.06	0.28	1.35	0.54–3.39	0.52
Uric acid-to-albumin ratio	<1 vs. ≥1	0.72	0.17–0.96	0.02	0.61	0.26–0.87	0.01
WBC		0.89	0.70–1.14	0.35	-	-	-
Hemoglobin		0.98	0.74–1.30	0.87	-	-	-
Creatinine		0.38	0.03–5.05	0.46	-	-	-
Uric Acid		1.55	1.12–2.13	0.08	-	-	-
Albumin		1.04	0.31–3.49	0.94	-	-	-

## Data Availability

The data presented in this study are available on request from the corresponding author. The data are not publicly available due to privacy or ethical restrictions.

## Abbreviations

The following abbreviations are used in this manuscript:

UAR	Uric Acid-to-Albumin Ratio
PFS	Progression-Free Survival
OS	Overall Survival
HR+	Hormone Receptor-Positive
HER2-	Human Epidermal Growth Factor Receptor 2-Negative
CDK4/6	Cyclin-Dependent Kinase 4/6
WBC	White Blood Cell Count
CRP	C-Reactive Protein
